# Optimization and evaluation of the Esperanza Window Trap to reduce biting rates of *Simulium damnosum sensu* lato in Northern Uganda

**DOI:** 10.1371/journal.pntd.0007558

**Published:** 2019-07-16

**Authors:** Denis Loum, Devon Cozart, Thomson Lakwo, Peace Habomugisha, Benjamin Jacob, Eddie W. Cupp, Thomas R. Unnasch

**Affiliations:** 1 Nwoya District Local Government, Nwoya, Uganda; 2 Center for Global Health Infectious Disease Research, College of Public Health, University of South Florida, Tampa, Florida, United States of America; 3 Vector Control Division, Ministry of Health, Kampala, Uganda; 4 The Carter Center, Uganda Office, Kampala, Uganda; Federal University of Agriculture, NIGERIA

## Abstract

**Background:**

Onchocerciasis, or river blindness, has historically been an important cause of blindness, skin disease and economic disruption in Africa and the Americas. It is caused by the filarial parasite *Onchocerca volvulus*, which is transmitted by black flies in the genus *Simulium*. Over the past decade, several international programs have been formed to control, or more recently eliminate onchocerciasis, using mass drug administration (MDA) of ivermectin. However, in many areas of Africa (particularly those which are endemic for the eyeworm, *Loa loa*, or where vector densities are very high) ivermectin MDA alone will not be sufficient to achieve elimination. In these situations, additional interventions may be necessary.

**Methodology/Principal findings:**

The Esperanza Window trap (EWT), a simple trap originally developed to replace human landing collections for entomological surveillance of *O*. *volvulus* transmission was optimized, resulting in a 17-fold improvement in trap performance. The optimized trap was tested in trials in schools and in agricultural fields to determine if it could reduce vector biting locally. The traps resulted in a 90% reduction in biting in the school setting. In the field setting, results varied. In one location, the traps reduced biting by roughly 50%, while in a separate trial, the traps did not significantly reduce the biting rate. Examination of the two settings suggested that trap placement may be critical to their success.

**Conclusions/Significance:**

These results suggest that the optimized EWT might be capable of reducing local vector black fly biting in areas commonly frequented by residents. Together with other recently developed methods of community directed vector control, the traps may augment ivermectin MDA, bringing the goal of onchocerciasis elimination within reach in much of Africa.

## Author summary

Onchocerciasis or river blindness is historically one of the most important causes of blindness and skin disease in the developing world. It is caused by infection with the filarial parasite *Onchocerca volvulus*. The finding that ivermectin was an effective and safe treatment for onchocerciasis and the decision by its manufacturer to donate it to treat this infection spawned the development of programs to eliminate river blindness through mass drug administration of ivermectin to the afflicted populations. This has dramatically reduced the prevalence of onchocerciasis worldwide and has resulted in its near elimination in the Americas. But ivermectin alone will not eliminate river blindness in much of Africa; additional interventions are necessary. Here we report the optimization of a simple trap for the black fly vector of *O*. *volvulus* and show that these traps can dramatically reduce vector biting in some settings. Together with other recently developed community directed methods of vector control, these traps may augment the effect of the ivermectin distribution programs, bringing the goal of elimination within reach in much of Africa.

## Introduction

*Onchocerca volvulus*, the causative agent of river blindness, remains endemic in most parts of Africa, despite mass drug campaigns with ivermectin spanning more than three decades [1, 2]. There are multiple causes for this entrenchment, many of which are the result of the difficulty in eliminating a vector-borne disease in general. Some are operational—for example inadequate drug coverage and/or poor timing of mass administration–while others are sociological, *i*.*e*., broad-scale apathy and occasional resistance on the part of individuals and communities to participate in treatment. A complicating factor throughout much of Central Africa is the co-endemicity of *O*. *volvulus* with *Loa loa*. The presence of the latter is particularly important because of severe adverse reactions that may occur in individuals with elevated *L*. *loa* microfilaremia following ivermectin treatment [3–5]. This has precluded the use of ivermectin mass drug administration (MDA) in many areas and may result in reduced community participation in areas co-endemic for loasis where ivermectin MDA is ongoing [6].

Aside from these operational issues, another fundamental problem is the long-standing hyperendemicity of *O*. *volvulus* associated with extremely high annual biting rates by the vectors, members of *Simulium damnosum sensu lato* species complex. Models show that vector abundance must be considered in situations where annual biting rates are high because treatment with ivermectin may not be able to achieve elimination on its own [7, 8]. Thus, there is a need for local vector control that is complementary to mass drug administration and is economical, ecologically benign and appropriate for much of rural Africa.

The Esperanza Window Trap (EWT) was originally developed as a tool to capture vector black flies to supplement or eventually replace human landing collections for verifying the interruption of transmission of *O*. *volvulus* in Latin America [9]. Its design was later modified for use in Africa [10], and shown to be an effective substitute for human landing collections in Uganda [11, 12]. The EWT, when deployed in households and in schoolrooms in rural Mexico was shown to be capable of significantly reducing the biting rate of the local vector *S*. *ochraceum*, suggesting that the EWT might have potential as a mechanism for vector control [13]. In the current investigation, we report the results of a series of studies aimed at optimizing the collection efficiency of the EWT in Uganda and report the results of studies evaluating the ability of the optimized trap to reduce biting of *S*. *damnosum* s.l. in two different settings. These studies were carried out in the Madi mid North focus of Uganda, which is the largest focus of onchocerciasis still active in Uganda. Elimination efforts here have lagged behind many of the other foci in Uganda due to political instability in the region and because the vector for *O*. *volvulus* in this area is *S*. *damnosum s*.*l*., which is less amenable to classical vector control measures than *S*. *neavei*, the most common vector in Uganda.

## Materials and methods

### Study sites

**Fig 1:**
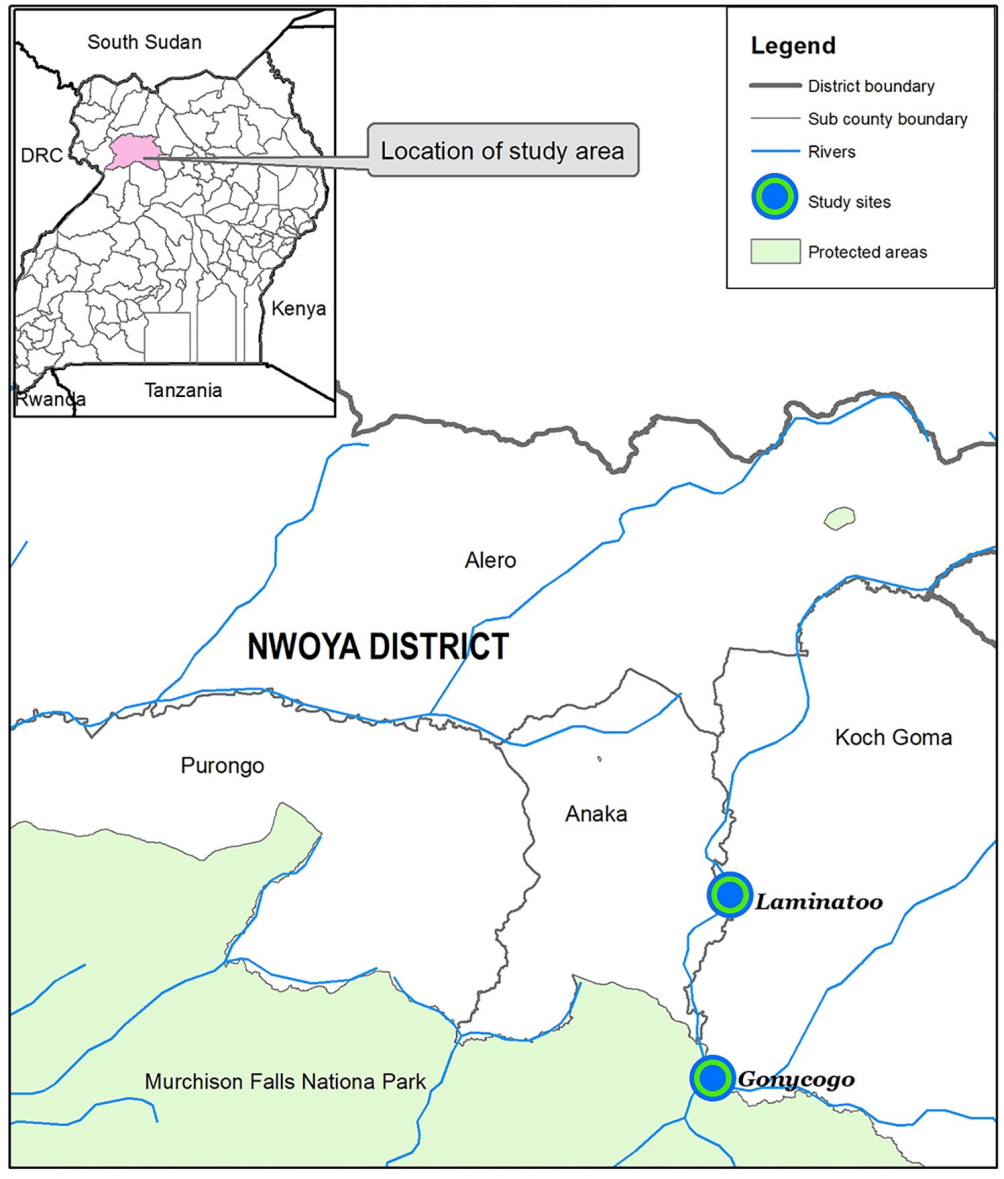
Location of Gonycogo and Laminatoo. This figure is reproduced from a previous publication describing the use of the EWT as a tool for entomological monitoring of *O*. *volvulus* transmission [12].

All experiments were carried out in the communities of Gonycogo and Laminatoo, located in the Nwoya district of Northern Uganda. Previous studies have indicated that vector density is high in these communities with individuals receiving 52 bites per day in Laminatoo and 100 bites per day in Gonycogo [12]. The location of these villages is shown in Fig 1. A detailed description of the study villages may be found in our previous publication [12].

The sibling species of *S*. *damnosum* s.l. present at these sites is the savanna dwelling cytospecies *S*.*damnosum sensu stricto*. The experiments were conducted from May, 2017 through January, 2019. All traps and landing collections were done on publicly accessible lands.

### Trap optimization studies

The performance of the different trap designs was evaluated in a pairwise fashion. Three pairs of sites were identified in each village where the traps were set up. The trap sites were at least 50m apart, to ensure that they were independent from one another. However, the traps were kept within 500m of the major black fly breeding site found near each community, to ensure that the traps were sampling from the same population of flies. One trap design was placed at one of each of the pair of sites, while the second design was placed at the second site in each pair. The traps were activated at 7AM by removing a sheet of plastic covering the surface of the trap, and the traps baited with a yeast sugar solution to produce CO_2_ and a dirty sock, as previously described [12]. The traps were permitted to operate until 6 PM, at which point the flies were removed from the trap surface by placing a small drop of white spirits on each fly to dissolve the glue and removing the fly from the trap with forceps. The flies were then placed in isopropanol, identified morphologically to species and the number of *S*. *damnosum s*.*s*. collected on each trap was recorded. Collections were carried out twice per week at all sites. The position of the trap designs in each pair of sites was switched weekly, to eliminate position effects. The number of trials ranged from 12 to 30, depending on the trial. The actual number of trials in each experiment is indicated in the figure legend for each pairwise comparison. Because the number of flies collected varied widely from day to day due to weather conditions, the daily collections from each of the designs evaluated in each study were normalized to the total number of flies collected from both trap designs on each day.

### School-based evaluation of the EWT as a localized vector control measure

These studies were conducted in two primary school classes in the village of Gonycogo, Koch in Goma sub-county in the Nwoya district. One of these classes was located in a thatched roofed building open to the outside on one side, while the second class was conducted in the open air under a tree. The school staff were instructed in the purpose of the study. The teachers then briefed the students on the purpose of the study, instructing them to refrain from touching or tampering with the traps. A human landing collector was placed at the periphery of the classes (so as not to disturb the children), and collections were carried out daily in the absence of the traps for a week to establish a baseline biting rate. The human landing collectors operated as a team of two individuals, which alternated collecting every hour throughout the day. Two traps were then set up near each of the classes and collections were continued for an additional week. Collections and traps were operated from 7AM to 5PM on weekdays when classes were in session. Collection numbers were normalized to those obtained from a human landing collection team located roughly 200m from the school, to control for variations in the overall fly population.

### Field-based evaluation of the EWT as a localized vector control measure

The studies to evaluate the ability of the EWTs to reduce biting rates in an agricultural setting were carried out in two types of fields in Laminatoo and Gonycogo, one planted with soybeans and one planted with tomatoes. The fields ranged in size from 0.5 to 1.3 hectares in area. A total of 5 traps were distributed around the periphery of each field and the intensity of vector biting monitored by a human landing collection team placed at the edge of the field. In addition to the collectors located in the test sites, an independent team was set up in each village located approximately 600 meters away from the fields to monitor any changes in the overall fly population. Collections were carried out every other day for a week (excluding Sunday) from 7AM through 6PM, at which point the traps were removed. Collections continued to be carried out in the absence of the traps for a week from 7AM through 6PM (excluding Sunday), at which point the traps were replaced. This pattern was continued for a total of five weeks.

### Statistical analysis

Fly collection numbers for all studies were recorded using whole day collections as the minimum time unit. To analyze the effect of trap size optimization, the number of flies caught by each trap was evaluated as a proportion of the number of flies caught that day. In the analysis of the unadjusted data, a one-sample Student’s T-test was used in order to determine whether the mean percentage of flies caught by the 1.5m traps was significantly different than 50%, i.e. accepting a null hypothesis that the 1.5m and 1m traps would be equally effective. In order to adjust for the effect of width, the null hypothesis was taken to be that the proportion of flies collected on the 1.5 m trap would be 60% of the total. This was based on the fact that the 1.5m trap made up 60% (1.5m/(1.5m + 1.0m)*100) of the total trap width tested in the trial. Similarly, in adjusting for area, the null hypothesis was taken that the 1.5m trap would collect 69% (2.25m^2^/2.25m^2^ + 1m^2^) of the flies.

To compare the mean fly capture counts between the 3 x 1m traps and 1.5m trap flanked by 2 x 1m traps, a Mann-Whitney test was used due to the non-normal distribution (Shapiro-Wilk, p = .029) of the data. A Student’s T-test was used in order to determine whether traps with a small black stripe would be more or less attractive when compared to traps consisting of three stripes of equal width. A nonparametric Mann-Whiney test was used for the analysis of stripe shape, due to the non-normal data distribution (Shapiro-Wilk, p = .005).

Multiple tests were used in the analysis of the data collected in the school study. An ANOVA test with a Dunnett post-hoc was used to determine whether there was any difference in the number of flies caught within the two classrooms and the outside human landing collector. Then, the fly counts within the classrooms were normalized to the number of flies caught by the external human landing collector. A Student’s T-test was then used to determine whether there was a significant difference in the normalized number of flies caught when there was a trap present compared to when there was no trap present.

The first step in analysis of the data collected to evaluate the effect of placing the traps in an agricultural setting was to normalize the field collection numbers to those of the external collector. The resulting data were then analyzed using a Mann-Whitney test, due to non-normal distribution (Shapiro-Wilk, p < .0001). Data from both villages were analyzed separately. Each analysis was conducted using a significance level of α = 0.05.

### Ethical clearance

The experiments described here were reviewed and approved by the Institutional Review Boards of the Uganda Vector Control Division (approval REF/VCDREC/071) and the University of South Florida (approval CR3_Pro00015108). All individuals participating in the study received Mectizan twice per year as part of the routine mass drug distribution program active in this area administered by the Uganda Ministry of Health.

## Results

### Optimization of the EWT platform

**Fig 2:**
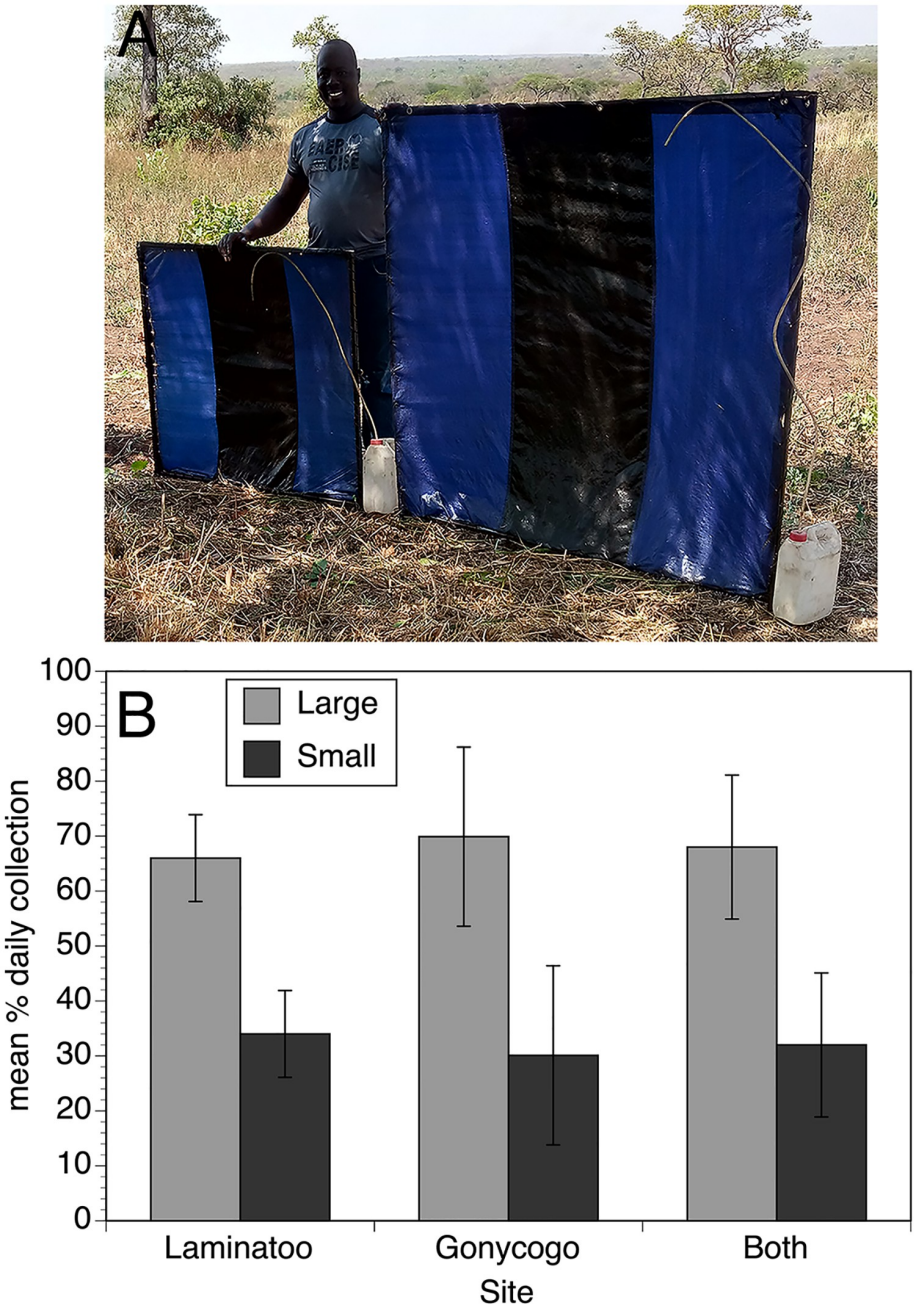
Relative performance of 1.5m and 1m square trap designs. Panel A: Photograph of the 1m and 1.5m trap designs. Panel B: Bars represent the relative performance of the 1m and 1.5m trap designs in Laminatoo, Gonycogo and data from both communities combined. Data are presented as the mean percentage of the total collection by day, normalizing for variations in the absolute number of flies collected on each day, as described in Materials and Methods. Error bars indicate the standard deviation associated with the means. The analysis from Laminatoo incorporated data collected from 18 paired trials, while the analysis from Gonycogo included 20 paired trials. Raw collection data for this study may be found in the S1 Table.

As a first step in optimizing the performance of the EWT, the effect of the size of the trap was evaluated. To accomplish this, two different trap sizes (1m square and 1.5m square; Fig 2, Panel A) were tested in a pairwise fashion, as described in Materials and Methods. The larger version of the trap was found to collect significantly more flies than did the smaller version (Fig 2, Panel B; p<0.0001).

**Fig 3:**
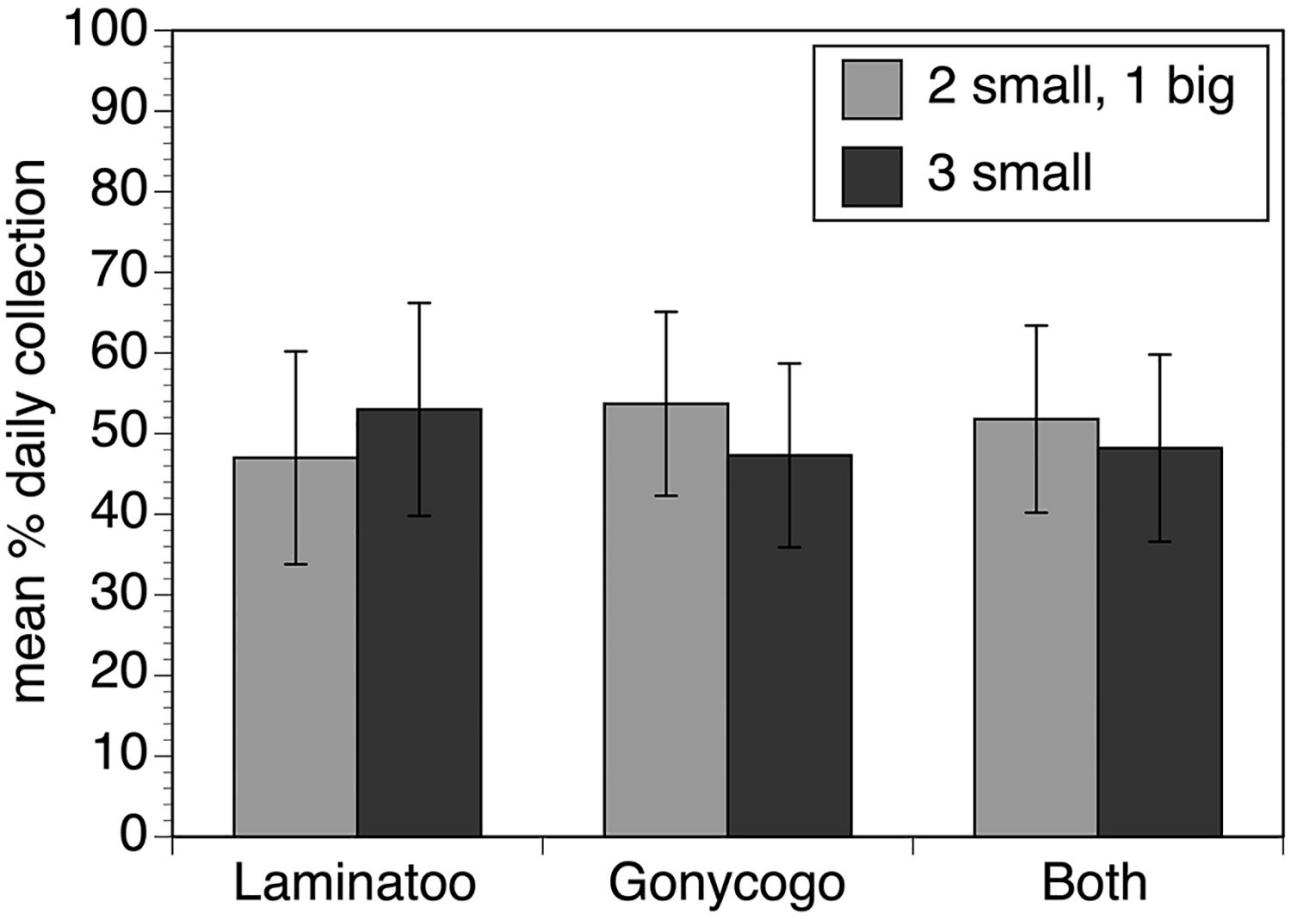
Relative performance of three trap batteries containing three 1m square traps versus two 1m square traps flanking a 1.5m square trap. Bars represent the relative performance of the different trap combinations in Laminatoo, Gonycogo and data from both communities combined. Data are presented as the mean percentage of the total collection by day, normalizing for variations in the absolute number of flies collected on each day, as described in Materials and Methods. Error bars indicate the standard deviation associated with the means. The analysis from Laminatoo incorporated data collected from 18 paired trials, while the analysis from Gonycogo included data collected from 24 paired trials. Raw collection data for this study may be found in the S2 Table.

The advantage conferred by the larger trap was maintained when analysis was adjusted for width (p< 0.005), but this advantage disappeared when analysis was adjusted for trap area (p = 0.65). Thus, the larger traps did not outperform their predicted performance based upon their area alone. This suggested that the increase in the collections seen by using the larger trap would not be any greater than if a number of smaller traps were deployed in an array. To test this hypothesis, a pairwise trial was conducted in which an array of three 1m traps was compared to an array consisting of two 1m traps flanking a single 1.5m trap. The collections obtained by the array of three 1m traps were not significantly different than those collected by the array containing the larger trap (Fig 3; p = 0.6). Because the larger traps were unwieldy and difficult to handle under field conditions, all subsequent trials used traps that were 1m square.

**Fig 4:**
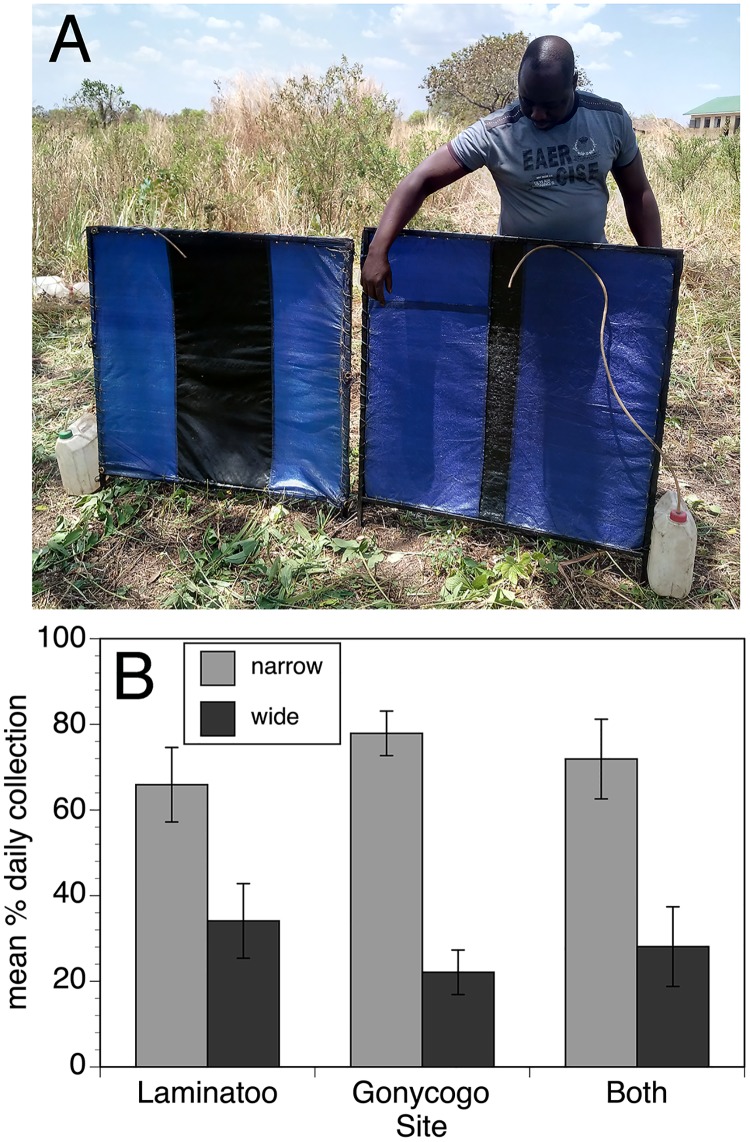
Relative performance of 1m traps with wide and narrow black stripes. Panel A: Photograph of the wide and narrow striped designs. Panel B: Relative performance of the wide and narrow striped trap designs in Laminatoo, Gonycogo and data from both communities combined. Data are presented as the mean percentage of the total collection by day, normalizing for variations in the absolute number of flies collected on each day, as described in Materials and Methods. Error bars indicate the standard deviation associated with the means. The analysis was conducted on data collected from 12 paired trials each in Laminatoo and Gonycogo. Raw collection data for this study may be found in the S3 Table.

The initial trials adapting the EWT for use in Africa demonstrated that a striped design was more effective than a solid blue color [10]. Subsequent studies suggested that traps containing one central blue stripe flanked by two black stripes, all of equal width, and traps containing two blue stripes flanking a single black stripe, all of equal width, were equally effective [11]. However, the effect of the width of the stripe had not been explored. To test this hypothesis, a pairwise comparison was carried out comparing the standard design with stripes of equal width to a design with a thinner black stripe (Fig 4, Panel A). In this case, the traps with the thin stripe collected significantly more flies than did the standard design (Fig 4, Panel B; p < 0.0001).

**Fig 5:**
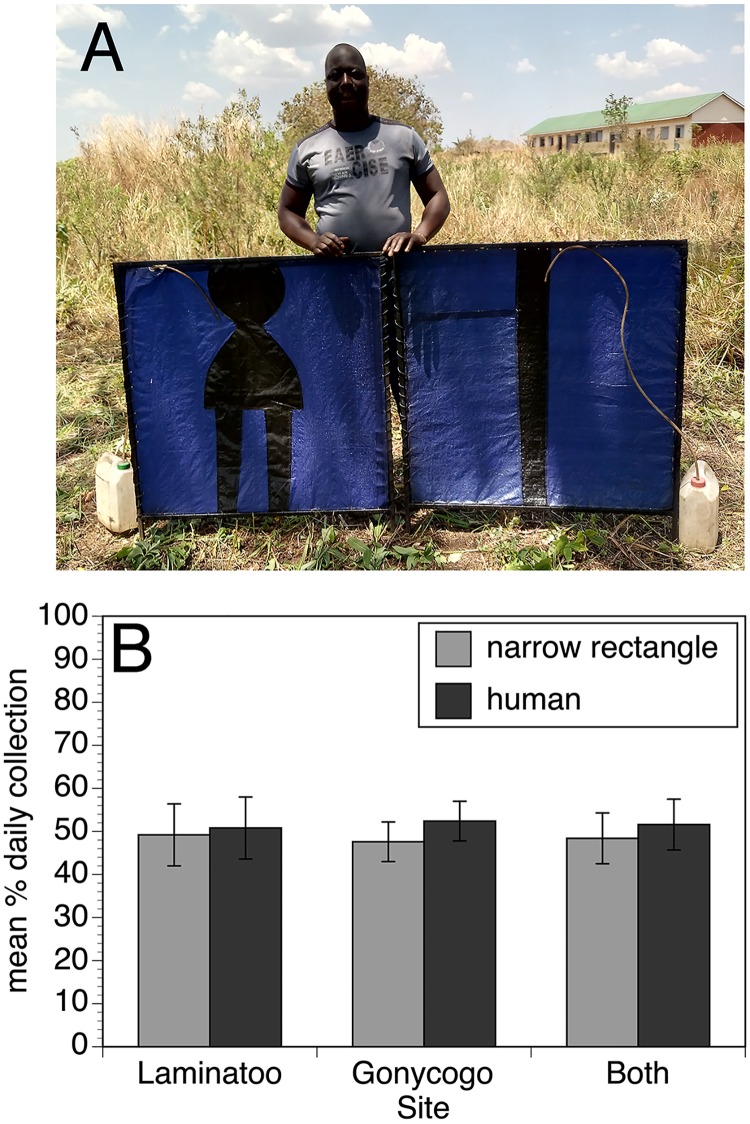
Relative performance of simple and human shaped stripe designs. Panel A: Photograph of the simple narrow stripe and human shaped stripe trap designs. Panel B: Relative performance of the simple narrow stripe and human shaped stripe trap designs in Laminatoo, Gonycogo and data from both communities combined. Data are presented as the mean percentage of the total collection by day, normalizing for variations in the absolute number of flies collected on each day, as described in Materials and Methods. Error bars indicate the standard deviation associated with the means. The analysis was conducted on data collected from 30 paired trials each in Laminatoo and Gonycogo. Raw collection data for this study may be found in the S4 Table.

As a final step in optimizing the EWT platform, the effect of changing the shape of the black stripe was investigated. In this trial, traps with a simple thin black stripe were compared to traps containing a central figure shaped like a human female (Fig 5, Panel A). It was found that the trap with the human shaped stripe did not collect significantly more flies than did the trap with the simple rectangular thin stripe (Fig 5, Panel B; p = 0.43).

Based upon these findings, all subsequent studies evaluating the EWT as a vector control measure utilized 1m square traps with a thin black stripe.

### Evaluation of the ability of the EWT to reduce biting of *S*. *damnosum s*.*l*

**Fig 6:**
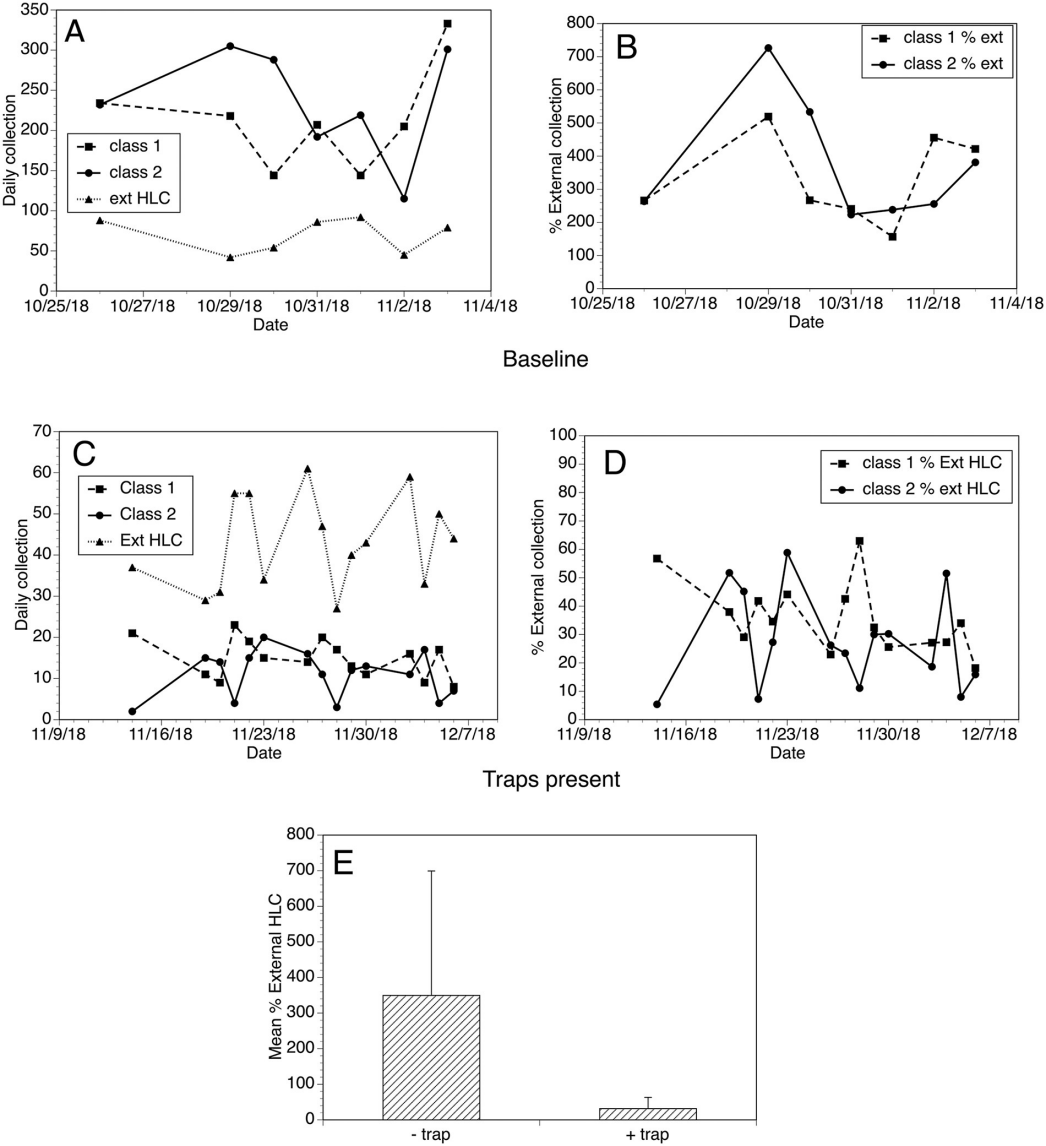
Evaluation of the ability of the optimized EWT to reduce biting in a school setting. The data were collected from two classrooms at Gonycogo Primary School and normalized for daily variations in the questing fly population using an external collector as described in Materials and Methods. Panel A: Daily collections obtained by the HLCs situated in the two classrooms and those obtained from the external collector in the absence of the traps. Panel B: Collections from the classrooms expressed as a percentage of the collection obtained by the external collector in the absence of the traps. Panel C: Daily collections obtained by the HLCs situated in the two classrooms and those obtained from the external collector in the presence of the traps. Panel D: Collections from the classrooms expressed as a percentage of the collection obtained by the external collector in the presence of the traps. Panel E: Mean collections obtained by the collectors in the classrooms normalized to those obtained by the external collector in the presence and absence of the traps. Bars represent the mean percentage of the collections obtained by the collectors relative to those obtained by the external collector in the presence and absence of the traps, and error bars the standard deviations of the normalized counts. Raw collection data for this study may be found in the S5 Table.

In studies conducted in Mexico, the Latin American version of the EWT when deployed in classrooms reduced biting by *S*. *ochraceum* by up to 50% [13]. Therefore, we decided to determine if the optimized version of the EWT described above would be capable of reducing biting in primary school classes in Uganda. In these studies, human landing collections were used to monitor biting rates in two open air classrooms in Gonycogo Primary School in the presence and absence of the traps. The numbers of flies collected in the classrooms were normalized to a human landing collection carried out 200m from the school, as a way of controlling for the day to day variation in the questing fly population. In the absence of the traps, the human landing collections in both classes were consistently higher than those of the external collector (Fig 6, Panels A and B). In the presence of the traps, this pattern was reversed—the classroom collections were consistently lower than those of the external collector (Fig 6, Panels C and D). When normalized for the number of flies collected by the external collector, the number of flies collected by the classroom collectors in the presence of the traps was on average 9% of those collected in the absence of the traps (Fig 6, Panel E; p < 0.0001).

**Fig 7:**
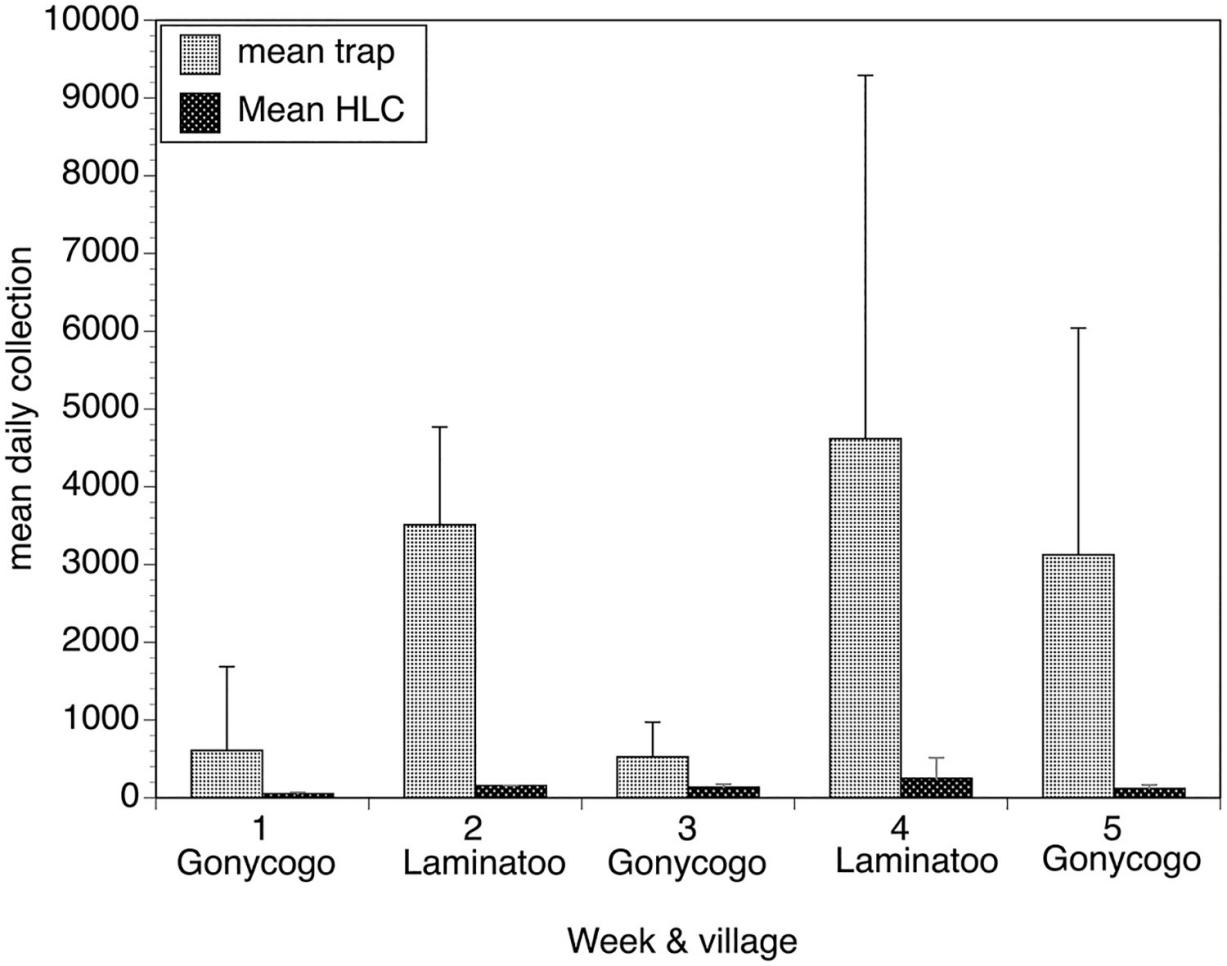
Daily collections obtained by traps and by the human landing collector located in the fields of Gonycogo and Laminatoo. Bars represent the mean daily collection obtained by each of the five individual traps situated around the two fields in each village and the mean collection obtained by the two HLCs situated in each field over the five weeks of the study. Error bars depict the standard deviation surrounding the mean daily collections. Data from each week were calculated from six days of human collection data and 30 days of trap collection data. Raw collection data for this study may be found in the S6 Table.

**Fig 8:**
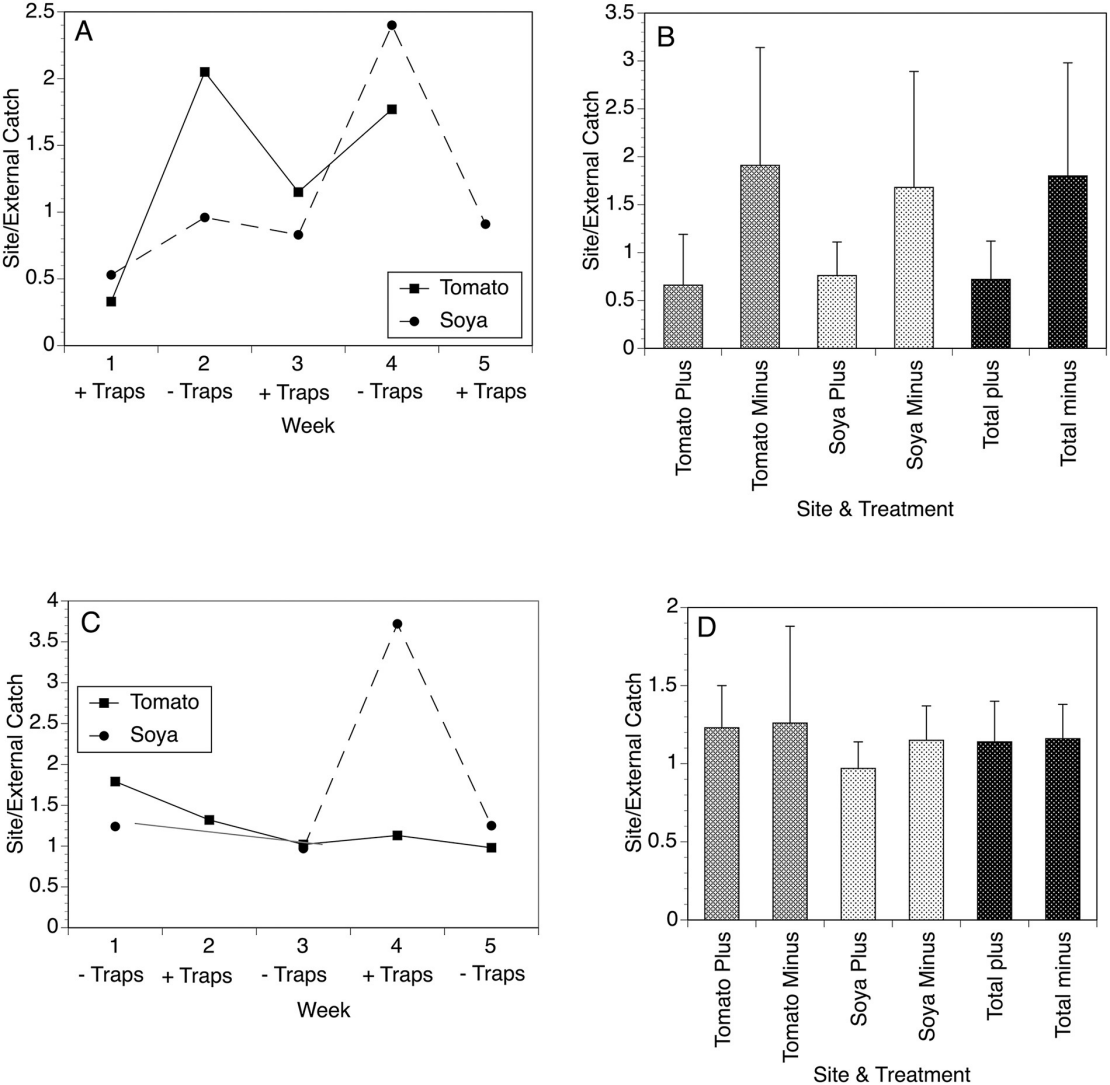
Effect of EWT trap deployment on biting rate in the fields of Gonycogo and Laminatoo. Panel A: Weekly fly collections in the tomato and soya fields of Gonycogo in the presence and absence of the traps. Traps were deployed and taken down in alternate weeks. Data are normalized to the number of flies collected by an external collector, to account for variations in the overall fly population. Panel B: Mean weekly collections in the tomato and soya fields of Gonycogo in the presence and absence of the traps. Error bars represent the standard deviations of the means. Panel C: Weekly fly collections in the tomato and soya fields of Laminatoo in the presence and absence of the traps. Traps were deployed and taken down in alternate weeks. Data are normalized to the number of flies collected by an external collector, to account for variations in the overall fly population. Panel D: Mean weekly collections in the tomato and soya fields of Laminatoo in the presence and absence of the traps. Error bars represent the standard deviations of the means. Raw collection data for this study may be found in the S6 Table.

The ability of the optimized EWT to reduce biting rates was also evaluated in two agricultural settings in Laminatoo and Gonycogo. In both villages, the traps collected large numbers of flies, with the mean fly collections per trap ranging from 4-fold to 26-fold greater than those collected by the associated human landing collector (Fig 7). Furthermore, in both the soya and tomato fields in Gonycogo, the number of flies collected in the fields by the human landing collector (when normalized to those collected by the external collector) was significantly less when the traps were present than when they were absent (p = 0.006; Fig 8, Panels A and B). In contrast, the biting rates in both fields in Laminatoo were not different when the traps were present when compared to when they were absent (p = 0.25; Fig 8, Panels C and D).

## Discussion

In the initial study carried out in Uganda that compared the EWT to human landing collections for surveillance of *O*. *volvulus* transmission, one EWT was found to collect roughly an equivalent number of flies as a human landing collector [12]. In the studies done in the soya and tomato fields of Gonycogo and Laminatoo, each optimized trap collected on average 17 times the number of flies collected by the HLC. The optimization process thus resulted in a dramatic improvement in the performance of the EWT. Despite this, it is likely that additional refinements to the trap might boost the trap performance even further. For example, earlier studies demonstrated that a synthetic bait formulation containing five compounds that are found in the sweat of most individuals previously shown to be attractive to *S*. *damnosum* s.l. [14] did not out-perform dirty socks worn by local residents of the study communities [12]. However, it is well known that individuals vary widely in their attractiveness to hematophagous insects [15, 16]. It is thus possible that the compounds found to be specific to, or in a higher concentration in the sweat of such highly attractive individuals might be used as a way to improve the baits for the EWT. Similarly, the use of alternative methods of CO_2_ generation [17], or carbohydrate sources other than sucrose for the generation of yeast produced CO_2_ [18] might improve trap performance.

The performance of the optimized traps varied widely. For example, Trap 3 in the study conducted in the soya field of Gonycogo generally out performed Trap 4; an extreme example of this occurred on May 17, 2018 when Trap 3 collected 5,000 flies and Trap 4 collected just 150 flies. This suggests that placement played a critical role in trap performance, which is consistent with studies carried out in Mexico [19]. Interestingly, the traps were placed in locations that appeared quite similar and all were in lightly shaded areas with good visibility on the edges of the fields. Furthermore, certain locations were not uniformly better than others. For example, trap 3 in the Gonycogo soya field out-performed trap 4 on all days but one, but on June 12, 2018, trap 4 collected 8,812 flies, while trap 3 collected just 1,006 flies. Thus, the performance of the traps varied widely from day to day, and the performance of the traps relative to one another was not consistent. This suggests that a trial and error process may be necessary to identify the locations that optimize trap performance.

The optimized EWT appeared to have potential for protecting groups of people from the bites of vector black flies. Before the traps were deployed in the school, the biting rate in both classrooms was significantly greater than that recorded by the external HLC. This in itself was somewhat disconcerting, as it suggests that the normal procedure used to site HLCs might actually underestimate the biting that people gathered in groups suffer. However, when the traps were deployed, the biting rate dropped dramatically in both classrooms. When normalized for the collection obtained by the external HLC, deployment of the traps reduced the biting rate in the classes by 91%. This reduction was greater than that observed in a similar study conducted in Mexico, where deployment of the traps in households and schools reduced biting by 14–51% [13]. This result suggests that the EWT might prove to be an effective means to reduce biting when deployed in key locations where people congregate for such daily activities as school, washing clothes and bathing.

In contrast to the dramatic reductions seen in the biting rates in the classrooms, the effects of deploying the EWTs in the fields were less clear. Overall, in Gonycogo a 60% reduction in the biting rate was observed when the traps were present when compared to when they were absent. However, the traps appeared to have no significant effect on the biting rate in the trials conducted in the fields in Laminatoo. One possible explanation for this difference might be in the location of the human landing collectors at each of the fields. In both communities, the human landing collectors were situated in partially shaded locations along the edge of the field. However, when examining satellite images of the fields, it was apparent that the collectors at both of the fields in Gonycogo were located on the western edge of the fields, while the breeding site was located to the east of the fields. Thus, flies coming from the breeding site would have passed the fields and the traps before encountering the collector. In contrast, the collectors in Laminatoo were located on the right edge of the field relative to the breeding site. Here, flies would not have had to travel past the traps before encountering the collector. This suggests that trap position might be very important in achieving a reduction in biting rates, with the traps best positioned so the flies are likely to encounter them before they encounter the humans the traps are protecting. More work will be necessary to test this hypothesis.

The trap design reported here was originally designed and optimized in areas endemic for the savanna dwelling species of *S*. *damnosum s*.*l*., *S*. *damnosum s*.*s*. and *S*. *sirbanum* [10]. It is possible that this design will not prove as effective in attracting other sibling species of the *S*. *damnosum s*.*l*. sibling species, such as those endemic to the forested or mountainous areas of Africa. Indeed, a recent evaluation of the EWT found that it was not very effective in attracting vector black flies in Tanzania [11], where *S*. *killibanum* and *S*. *nkusi* are the predominant vectors [20]. Thus, optimization studies like those reported here may need to be conducted in areas where initial trap performance is not satisfactory. A second limitation to the widespread application of the EWT is that the Tangle Trap glue is not locally available in Africa and needs to be imported. Identifying a locally available replacement for Tangle Trap would make widespread application of the traps more likely, as all other components of the trap are inexpensive and available throughout Africa.

Recently, we reported the evaluation of an alternative community-based method of vector control, removal of streamside vegetation along the *S*. *damnosum s*.*l*. breeding sites. This so called “slash and clear” method resulted in dramatic (>90%) reductions in biting rates that lasted for up to three months after the intervention [21]. It is possible that the application of the slash and clear process together with the deployment of EWTs in selected areas where biting rates are high might result in an almost complete elimination of vector biting. For example, if the effects of the two methods are merely additive, one might hypothesize that combining slash and clear with selective deployment of the EWTs could result in 94–99% reductions in the *S*.*damnosum s*.*l*. biting rate. Apart from the relief provided to the communities from the biting nuisance, this reduction would be expected to accelerate the elimination of *O*. *volvulus* transmission. Furthermore, the EWTs might prove to be a good alternative method of community directed vector control in areas where slash and clear cannot be easily applied (e.g., along very large and dangerous rivers or in areas where the breeding sites are too small to be easily targeted by slash and clear). Studies investigating the effect of combining EWTs with slash and clear are currently underway.

## Supporting information

S1 Table Raw collection data from the comparison of 1m^2^ and 1.5m^2^ traps. (XLSX)

S2 Table Raw collection data from the comparison of three 1m^2^ traps with one 1.5m^2^ trap flanked by two 1m^2^ traps. (XLSX)

S3 Table Raw collection data from the comparison of wide and narrow black stripe versions of the 1m^2^ trap. (XLSX)

S4 Table Raw collection data from the comparison of human shaped and narrow black stripe versions of the 1m^2^ trap. (XLSX)

S5 Table Raw collection data from the school-based study of the effect of the traps on biting rates. (XLSX)

S6 Table Raw collection data from the field-based study of the effect of the traps on biting rates. (XLSX)
